# A randomized clinical trial to assess the effect of zinc and vitamin D supplementation in addition to hypertonic saline on treatment of acute bronchiolitis

**DOI:** 10.1186/s12879-022-07492-2

**Published:** 2022-06-13

**Authors:** Parisa Khoshnevisasl, Mansour Sadeghzadeh, Koorosh Kamali, Armita Ardalani

**Affiliations:** 1 Social Determinants of Health Research Center, Zanjan University of Medical Sciences, Zanjan, Iran; 2 Metabolic Disease Research Center, Zanjan University of Medical Sciences, Zanjan, Iran; 3 Department of Pediatrics, School of Medicine, Zanjan University of Medical Sciences, Zanjan, Iran

**Keywords:** Bronchiolitis, Infant, Supplements, Vit D, Zinc

## Abstract

**Background:**

Bronchiolitis, the most common cause of hospitalization in infancy has not yet a definitive treatment. This study was conducted to assess the effect of Zinc and vitamin D on treatment of infants with bronchiolitis.

**Methods:**

In this double blind, randomized clinical trial, 94 infants aged 2 to 23 months, admitted in Mousavi Hospital in Zanjan, Iran, with the diagnosis of acute bronchiolitis were randomly assigned into 3 groups. The control group was only treated with hypertonic saline. The two case groups received either 100 unit/kg/day of Vitamin D or 20 mg/day of zinc in addition to hypertonic saline. Wheezing, duration of hospital stay, cough, cyanosis, respiratory distress and the respiratory rate in the first, third and seventh day of hospitalization were evaluated.

**Results:**

There was no significant difference between groups in terms of age, sex, weight, passive smoking, wheezing, oxygen saturation, cyanosis and type of delivery. On the third day of hospitalization, the respiratory rate/min in the control group, the groups receiving vitamin D and zinc were 45.2 ± 10.7, 37.8 ± 3.9 and 41.1 ± 9.1 respectively and the result of repeated measure analysis didn’t show any significant difference between the 3 groups (P = 0.562). Duration of hospitalization in the group receiving Vitamin D or zinc and in controls were 4.2 ± 2.6, 4.4 ± 2.2 and 5.1 ± 2.4 days respectively and this difference was not significant. Zinc receiving patients did not differ from the control group regarding to respiratory rate, cyanosis and wheezing.

**Conclusion:**

Vitamin D or zinc administration was not effective in reducing respiratory rate in children with bronchiolitis.

*Trial registration* This project was approved by the Institutional Ethics Committee (IR, ZUMS.REC.1396.50), and registered on IRCT (IRCT20131217015835N7).

## Background

Acute bronchiolitis is the result of the obstruction of lower respiratory tract [1] caused by a viral infection [2]. It is one of the most important diseases of children less than two years old and the most common cause of hospitalization due to viral lower respiratory tract infection in infancy [3]. The most common symptoms are cough, wheezing, tachypnea and hyperinflation [1]. Respiratory syncytial virus (RSV) and rhinoviruses (RV) are the two major etiologies of the disease [1]. Unfortunately, there isn’t still a definitive treatment for the disease and only supportive care is recommended [2, 3]. Bronchodilators, corticosteroids, antiviral agents and antibiotics are not recommended in these patients. Although there are controversial results about hypertonic saline, it seems that the benefit of this treatment is seen when the hospital stay exceeds 72 h. Airway suctioning and hydration are the most suggested therapies [2].

Recently in the literature, some supplementations are mentioned to improve respiratory diseases and immunity. It is thought that vitamin D has immunomodulatory effects and its deficiency causes a variety of respiratory infections such as bronchiolitis, otitis media and tuberculosis [4]. Moreover, vitamin D level of cord blood was related to lower respiratory tract infections and is important in lung development [5].

On the other hand, zinc is important in immune function by supporting mucous membranes [6], blocking the virus entry [7], increasing ciliary beat frequency [8] and improving ciliary length of bronchial epithelium [9]. Some studies showed that lower respiratory tract infections were related with low serum zinc levels [10]. It was concluded that vitamin D and zinc may have beneficial effects in the treatment of bronchiolitis.

Due to the small number of studies assessing the role of these supplements in the treatment of this disease, this clinical trial was conducted to evaluate the effect of Zinc and vitamin D on treatment of infants with bronchiolitis.

## Methods

In this double blind, parallel (allocation ratio 1:1), randomized clinical trial, 90 infants aged 2 to 23 months, who were admitted to Mousavi Hospital in Zanjan, Iran, from May 2018-May 2019, with the diagnosis of Acute Bronchiolitis, were evaluated for the effect of zinc or Vit D supplementation on their treatment.

This project was approved by the Institutional Ethics Committee (IR, ZUMS.REC.1396.50), and registered on IRCT (Iranian Registry of Clinical Trials); (IRCTID: IRCT20131217015835N7) and the full date of first registration is 17/05/2018.

The sample size was calculated based on previous study [11] assuming type 1 error 0.5, power 0.8 and treatment efficacy 25% and each group contained 30 patients.

The first wheezing attack in children 2 to 23 months old with lower respiratory tract infection in lack of any cause for wheezing was considered as bronchiolitis. The diagnosis of acute bronchiolitis was made according to the clinical examination performed by a pediatrician.

### Inclusion and exclusion criteria

Inclusion criteria consisted of the age between 2 to 23 months, the first admission of viral respiratory infection associated with wheezing, pulse rate less than 180, respiratory rate less than 100.

The exclusion criteria were: History of previous wheezing, prematurity, aspiration, cardiopulmonary disease, neuromuscular disease, immune deficiency, need for mechanical ventilation, drug intolerance, side effects of drugs, previous use of corticosteroid or bronchodilators in the last 2 weeks and use of zinc or vitamin D more than routine from 1 month before admission. It should be noted in our country, infants routinely get 400 units/day of vitamin D provided by health care centers for free. Patients who needed PICU care were also excluded from the study.

After obtaining written consent from parents, patients were randomly assigned in three groups: Two intervention groups receiving Zinc or Vitamin D, and the third group (control group) that did not take any additional supplement.

### Randomization

Patients were assigned for interventions and control groups based on simple and individual randomization by a statistician who was not involved in the treatment by using random number table. For allocation concealment sequentially numbered, sealed, opaque envelopes were used. In this trial, participants and the outcome evaluators were blinded. Participants knew that they may take supplements besides routine treatment, but instead of the name of the supplements we used codes. The investigators evaluated the outcomes according to the codes.

### Treatment protocol

All groups received hypertonic saline for treatment of bronchiolitis. In the first group 20 mg of Zinc Sulfate provided by Pakhsh Mahya Company Iran was given every day for a week. The second group received 100 units per kg of Vitamin D provided by Vitabiotik Pakhsh Company Iran, daily for a week. The control group received only hypertonic saline, no supplement was given to this group. Both supplements were labeled with the randomization codes and given by a nurse who was not involved in the study.

### Outcome measures

The primary outcome was recovery of wheezing, respiratory distress and cyanosis in the first, third and seventh day in the hospital. The secondary outcome was the duration of hospital stay.

The outcomes were evaluated by the involved physician at the beginning, third and seventh (if the patient still was hospitalized) days of hospital stay.

Demographic data including age, sex, weight, maternal age, delivery method and possible confounding factors including passive smoking as well as side effects of medications and patient adherence were recorded in premade questionnaire.

### Data analysis

Data were analyzed by SPSS software version 16.0 (SPSS Inc, Chicago, USA). Descriptive data are presented as number, percent, mean and standard deviation. Qualitative variables were analyzed by chi-square test, Fisher exact test. Quantitative variables (had normal distribution) were analyzed by independent sample t-test, one way and repeated measures analysis of variance (ANOVA). P value was calculated in each group. Statistically significant level was considered less than 0.05.

## Result

**Fig. 1:**
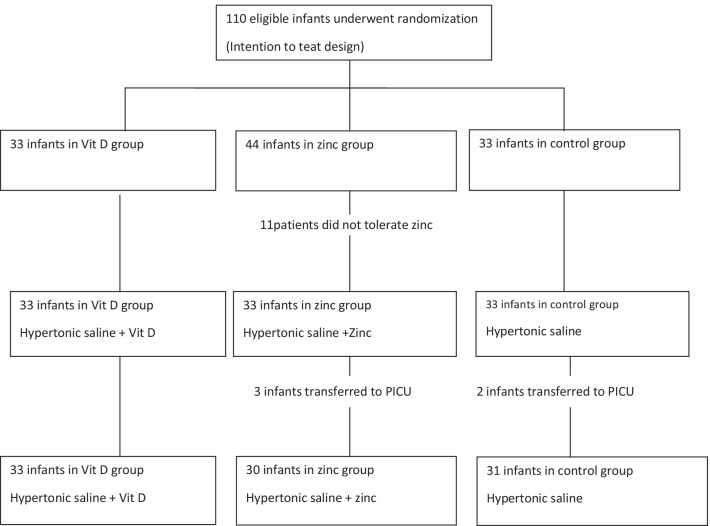
Flow chart of the study design

From May 2018-May 2019, 94 infants from 110 eligible patients were randomly assigned to three groups, including two intervention groups and a control group. Vitamin D was administered to thirty-three infants who had no side effects or intolerance, and none were excluded. Forty four patients received zinc sulfate, of which 11 people did not tolerate the drug and thus 33 infants were included in the zinc receiving group. Thirty three patients in the control group did not receive any supplementation. Three infants of zinc receiving group and 2 patients from control group were transferred to PICU due to respiratory distress and were excluded. Finally, a comparative study was performed between a group of 33 patients receiving vitamin D, a group of 30 infants receiving zinc and a group of 31 patients in the control group who did not receive any supplements. Flow chart of the study is shown in Fig. 1 and the demographic data are shown in Table 1.

**Table 1:** Demographic characteristics of patients in interventions and control groups

Variable	Zinc group n (%)	Vit D group n (%)	Control group n (%)	P value
Sex				
Female	16 (53.3%)	9 (27.3%)	12 (38.7)	0.106
Male	14 (46.7%)	24 (72.7%)	19 (61.3)	
Type of delivery				
Vaginal	17 (56.7)	18 (54.5)	13 (41.9)	0.416
Cesarean section	13 (43.3)	15 (45.5)	18 (58.1)	
Passive smoker				
Yes	9 (30)	16 (48.4)	14 (45.2)	0.336
No	21 (70)	17 (51.6)	17 (54.8)	
Age (month: mean ± sd)	9.5 ± 6.9	10.2 ± 12.1	10.7 ± 9.8	0.878
Weight (Kg: mean ± sd)	8.1 ± 2.1	8.5 ± 3.4	8.4 ± 2.4	0.881
Maternal age (year: mean ± sd)	26.6 ± 6.5	26.4 ± 6.0	28.5 ± 6.0	0.343
Oxygen saturation	92.3 ± 3.8	89.7 ± 9.5	92.8 ± 2.5	0.548

In the zinc receiving group, 16 girls and 14 boys with the mean age of 9.5 ± 6.9 months, and in the Vit D receiving group 9 girls and 24 boys with the mean age of 10.2 ± 12.1 months entered the study. The control group consisted of 12 girls and 19 boys with the mean age of 10.7 ± 9.8 months. As seen in the Table 1 there were no significant difference between the three groups in terms of gender, type of delivery, passive smoking, age, weight, maternal age and oxygen saturation.

The outcomes of the study are compared in Table2.

**Table 2:** Comparison of study groups regarding outcomes

Variables			Zinc group N (%)	Vit D group N (%)	Control group N (%)	P value
Respiratory distress	First day	Yes	21 (70.0)	25 (75.8)	20 (64.5)	0.617
		No	9 (30.0)	8 (24.2)	11 (35.5)	
	Third day	Yes	9 (39.1)	3 (13.6)	11 (40.7)	0.086
		No	14 (60.9)	19 (86.4)	16 (59.3)	
	Seventh day	Yes	1 (16.7)	0 (0.0)	3 (42.9)	0.259
		No	5 (83.3)	6 (100.0)	4 (57.1)	
Wheezing	First day	Yes	30 (100.0)	33 (100.0)	31 (100.0)	1.000*
		No	0 (0.0)	0 (0.0)	0 (0.0)	
	Third day	Yes	8 (34.8)	8 (36.4)	9 (33.3)	0.976
		No	15 (65.2	14 (63.6)	18 (66.7)	
	Seventh day	Yes	0 (0.0)	0 (0.0)	3 (42.9)	0.077*
		No	6 (100.0)	6 (100.0)	4 (57.1)	
Cyanosis	First day	Yes	3 (10.0)	5 (15.2)	5 (16.1)	0.758
		No	27 (90.0)	28 (84.8)	26 (83.9)	
	Third day	Yes	0	0	0	1.000*
		No	23 (100.0)	22 (100.0)	27 (100.0)	
	Seventh day	Yes	0	0	0	1.000*
		No	6 (100.0)	6 (100.0)	7 (100.0)	
Respiratory rate	First day		46.4 ± 10.4	48.3 ± 13.9	52.3 ± 12.9	0.002** 0.562***
	Third day		41.1 ± 9.1	37.8 ± 3.9	45.2 ± 10.7	
	Seventh day		38.2 ± 7.3	38.5 ± 3.6	43.3 ± 9.5	
O2 saturation	First day		92.3 ± 3.8	89.7 ± 9.5	92.8 ± 2.5	0.548***
	Third day		95.7 ± 2.7	94 ± 3.5	97 ± 0.7	0.320***
	Seventh day		96.2 ± 0.7	95.8 ± 0.8	97 ± 0.9	0.561***
Duration of hospitalization			4.4 ± 2.2	4.2 ± 2.6	5.1 ± 2.4	0.326

*P values are calculated by Fisher exact test

** Within group P value by repeated measures ANOVA analysis

*** Between groups P value by repeated measures ANOVA analysis

At the first day of hospitalization, in all three groups, there was no significant difference regarding the number of infants with respiratory distress. On the third day of hospitalization, most of patients had not respiratory distress in all three groups and there was no significant difference between groups (P value = 0.086). On the seventh day in all three groups, respiratory distress decreased and there was no significant difference between the groups (P = 0.259).

At the beginning of the study, all patients in all three groups had wheezing. On the third day, wheezing remained in36.4% of the group receiving Vit D, 34.8% of patients who received zinc and in 33.3% of the control group. On the seventh day, in the control group 3 infants (42.9%) had still wheezing. Although in both intervention groups all infants were completely recovered, no significant difference was found between the study groups in the duration of wheezing.

At the first day, 5 patients (16.1%) in the control group, 5 patients (15.2%) in the vitamin D group and 3 patients (10.0%) in the zinc recipient group had cyanosis and there was not a significant difference between groups. On the third and seventh days, none of the patients were cyanotic.

According to Table 2, at the beginning of the hospitalization, there was no significant difference between the three groups regarding to the respiratory rate (P = 0.175). On the third day, the respiratory rate in the control group, the group receiving vitamin D and zinc were 45.2 ± 10.7/min, 37.8 ± 3.9/min and41.1 ± 9.1/min respectively. No significant difference was found in the respiratory rate of the three groups in the seventh day (P = 0.390). Although by repeated measures ANOVA analysis in each group the respiratory rate was significantly decreased from the first day to seventh day (P = 0.002), no significant difference between the 3 groups was shown (P = 0.562).

All patients in all three groups had cough at the beginning of the study. On the third day, 24 (88.9%) infants in the control group,15 (68.2%) patients in the group receiving vitamin D and 21 (91.3%) patients in the group receiving zinc, had cough (P value = 0.071). On the seventh day, 6 patients in the control group (75.0%), 3 patients in the vitamin D group (50.0%) and 4 patients (66.7%) in the zinc receiving group had cough (P value = 0.837). These differences were not significant.

According to Table 2, no significant difference was found in the length of hospital stay between groups (P = 0.326) and O2 saturation in the treatment groups.

No side effects of the drugs were found before the seventh day.

## Discussion

In this study, the primary outcomes (the duration of respiratory distress, respiratory rate, wheezing, cyanosis,) and the secondary outcome (the length of hospitalization) of patients with bronchiolitis were evaluated. On the third and seventh day of hospitalization, we did not find a significant difference in the primary and secondary outcomes between the study groups.

In our study, children treated with zinc did not differ from the control group in respiratory rate, cyanosis, wheezing and hospital stay. In a clinical trial conducted at Mashhad by Heidarian et al. on 50 infants aged 2 to 23 months in 2 control and zinc receiving groups, no significant difference in improving clinical manifestations of acute bronchiolitis was found between two groups [12]. This result is consistent with our study. Malik et al. showed that zinc supplements for 2 weeks did not prevent acute lower respiratory tract infections in 6–11 months old infants [13]. Coles suggested that the prophylactic effect of zinc is different in diverse etiologies [14]. Somé et al. mentioned that zinc prophylaxis did not reduce lower respiratory tract infections [15]. Similarly Srinivasan et al. stated that although zinc has decreased the fatality of pneumonia, it did not affect respiratory rate and oxygen saturation of these patients [16]. Moreover, zinc supplementation did not decrease hospitalization stay on children with pneumonia in Tanzania [17].

On the other hand, numerous sources have been written about the positive effects of zinc on the immune system and its effect on lower respiratory tract infections. Ibraheem et al. concluded that children with acute lower respiratory tract infections had low serum zinc level in Nigeria [10]. Most of studies with positive effect of zinc are conducted on bacterial pneumonia and when omitting the infants with wheeze due to bronchiolitis and viral disease, the effect of zinc was greater [18].The different response of zinc in bronchiolitis, is due to fact that this trace element can be beneficial and support the immune system after more than 100 h of illness [19] which may not be possible in this acute viral disease. Also, at the beginning of the infection, zinc is shifted to the liver and therefore cannot have its beneficial effects [19].

Many studies have shown significant low levels of vitamin D in patients with bronchiolitis [20–23]. Golan-Tripto et al. stated that patients with bronchiolitis had a lower serum D3 compared to patients with febrile non respiratory diseases [20] and Vo et al. showed that patients with lower serum D3 had an increased risk of hospitalization for bronchiolitis [24]. Meanwhile, there are some studies showing that there is no relationship between vitamin D levels and bronchiolitis, [5, 25, 26]. Perhaps the reason for this difference may be the dissimilarity in sunlight exposure and supplement consumption. We found few clinical trials studying the effect of vitamin D on this disease.

The trial study of Saad et al. in Egypt on 90 infants with bronchiolitis showed that vit D receiving group had a significant improvement of bronchiolitis compared with controls [27]. Although in our study, the group receiving vit D had decreased respiratory rate and improved faster but the difference was not significant. The duration of hospitalization in the study of Saad et al. was also decreased. In our results the hospitalization stay was shorter in vit D3 supplementation group compared to controls but the difference was not significant.

The role of vitamin D in the innate immune system is thought to be due to induction of defensins and cathelicidin which are important in the defense against bacteria and viruses in respiratory tract [20, 28, 29] as well as neutrophils and macrophages [27]. Cathelicidin level in skin is elevated after UV exposure. In winter the lesser amount of UV leads to reduced Cathelicidin and therefore increased infections [20]. This may be one of the reasons why bronchiolitis is more prevalent in winter. Moreover a significant relationship between the severity of bronchiolitis and vitamin D receptor polymorphism [30] as well as vit D levels in patients was shown [24].

It seems that because of short term involvement of bronchiolitis, the effect of zinc or vit D administration cannot be seen in this short period of disease.

Our study compared also zinc with vitamin D supplementation and showed that although vit D receiving group had lesser respiratory rate and distress on third day, this result was not significant. We did not find any trial comparing these two supplementations but the better effect of vitamin D although not significant, can be explained by the fact that Iranian children had low levels of vit D [31] and that zinc has not a significant effect on acute viral infections [18].

This study has some limitations. We did not have the serum levels of zinc and vitamin D before and after the intervention. Also we think that we might have more clear results with a greater sample size.

## Conclusion

The data of this study does not support the benefit of vit D or zinc supplementation on treatment of acute bronchiolitis, which may be due to the short-term nature of the disease but we also emphasize that conducting multicentric clinical trials with greater sample size can dispel any doubts.

## Data Availability

All data and materials are available and potentially shareable on request. The in charge person is Dr. Mansour Sadeghzadeh. The phone number is + 989,121,757,737 and the email is sadeghzadeh@zums.ac.ir.

## Abbreviations

IRCT: Iranian Registry of Clinical Trials
PICU: Pediatric Intensive Care Unit
RSV: Respiratory syncytial virus
RV: Rhinoviruses
SD: Standard Deviation
UV: Ultraviolet
